# Supplementary material for: Basal-Type Breast Cancer Stem Cells Over-Express Chromosomal Passenger Complex Proteins
Source: Cells. 2020 Mar 13;9(3):709. doi: 10.3390/cells9030709 (PMC7140627; doi:10.3390/cells9030709)

Basal-type Breast Cancer Stem Cells over-express chromosomal passenger complex proteins.

Angela Schwarz-Cruz_y_Celis, et. al.

Supplementary Figure 1. Enriched signaling cascade of gene isoforms upregulated in breast Cancer Stem Cells. the -log p value for the gene ratio is shown(lines). In addition, the z score, which reflects the direction of the activation is shown in the bars. Data generated using the Ingenuity Pathway Analysis (IPA) suite.

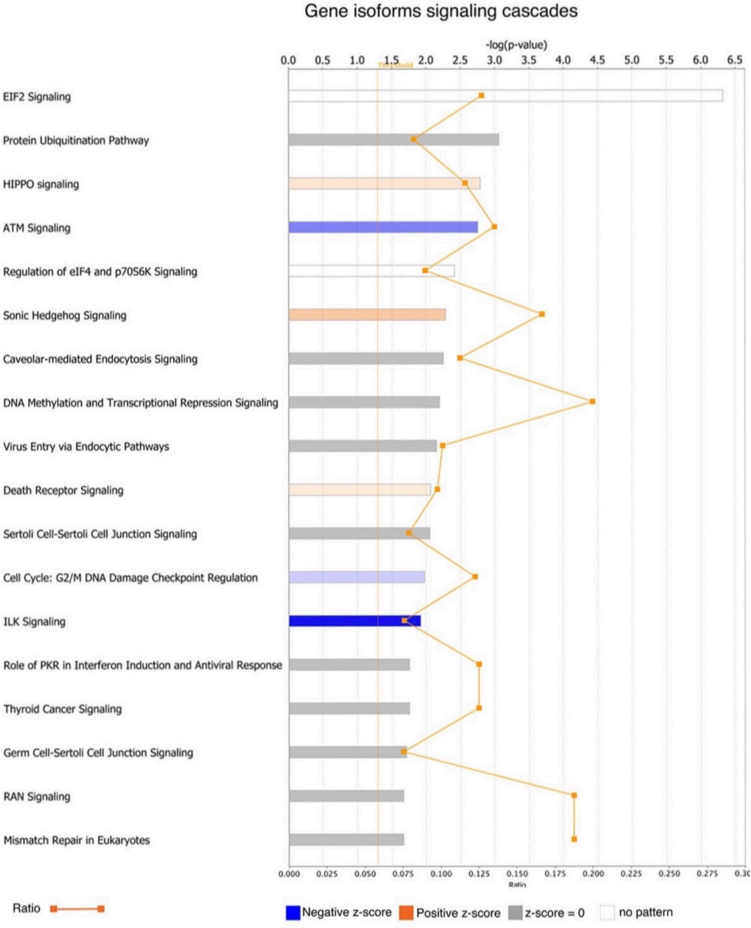

Supplement: Supplementary file 1 [file cells-09-00709-s001.zip › supple-proofreading/Figure S1.pdf]
