# Supplementary material for: Basal-Type Breast Cancer Stem Cells Over-Express Chromosomal Passenger Complex Proteins
Source: Cells. 2020 Mar 13;9(3):709. doi: 10.3390/cells9030709 (PMC7140627; doi:10.3390/cells9030709)

Basal-type Breast Cancer Stem Cells over-express chromosomal passenger complex proteins.

Angela Schwarz-Cruz_y_Celis, et. al.

Supplementary Figure 2. Kaplan-Meier survival plot for relapse-free and overall survival in breast cancer patients molecular subtypes, classified with the chromosomal passenger protein module. Data were derived from a group of previously published cohorts using kmplot [1]

1. Lanczky A, Nagy A, Bottai G, Munkacsy G, Szabo A, Santarpia L, et al. miRpower: a web-tool to validate survival-associated miRNAs utilizing expression data from 2178 breast cancer patients. Breast Cancer Res Treat 2016;160(3):439-446.

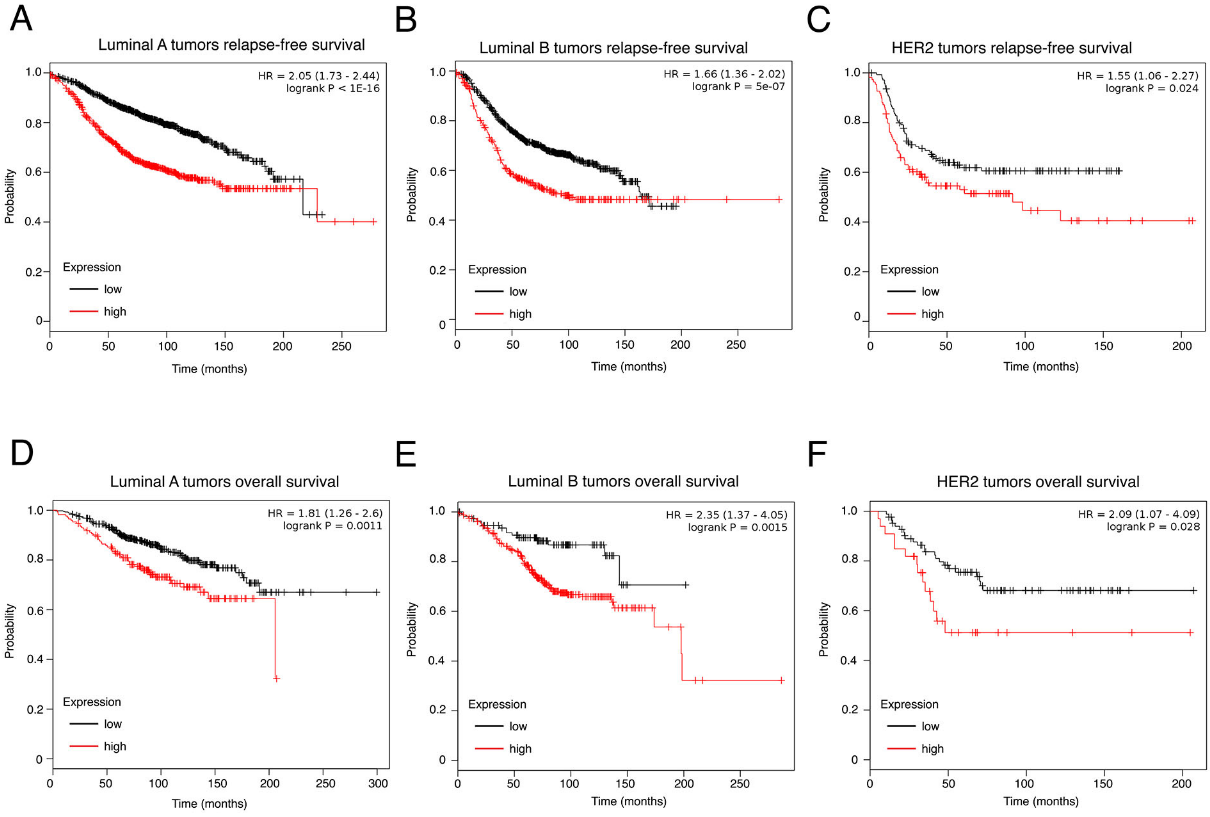

Supplement: Supplementary file 1 [file cells-09-00709-s001.zip › supple-proofreading/Figure S2.pdf]
