# Supplementary material for: Basal-Type Breast Cancer Stem Cells Over-Express Chromosomal Passenger Complex Proteins
Source: Cells. 2020 Mar 13;9(3):709. doi: 10.3390/cells9030709 (PMC7140627; doi:10.3390/cells9030709)

Basal-type breast cancer stem cells over-express chromosomal passenger complex proteins

Angela Schwarz-Cruz y Celis, et. al.

Supplementary Figure 3. Kaplan-Meier survival plot for relapse-free and overall survival in breast cancer patients. (A, C, E) Kaplan-Meier survival plot for relapse-free survival in breast cancer patients classified individually for each CPPM gene: (A) BIRC5, (C) AURKB and (E) INCENP. (B, D, F) Kaplan-Meier survival plot for overall survival in breast cancer patients, classified individually for each CPPM gene: (B) BIRC5, (D) AURKB and (F) INCENP.

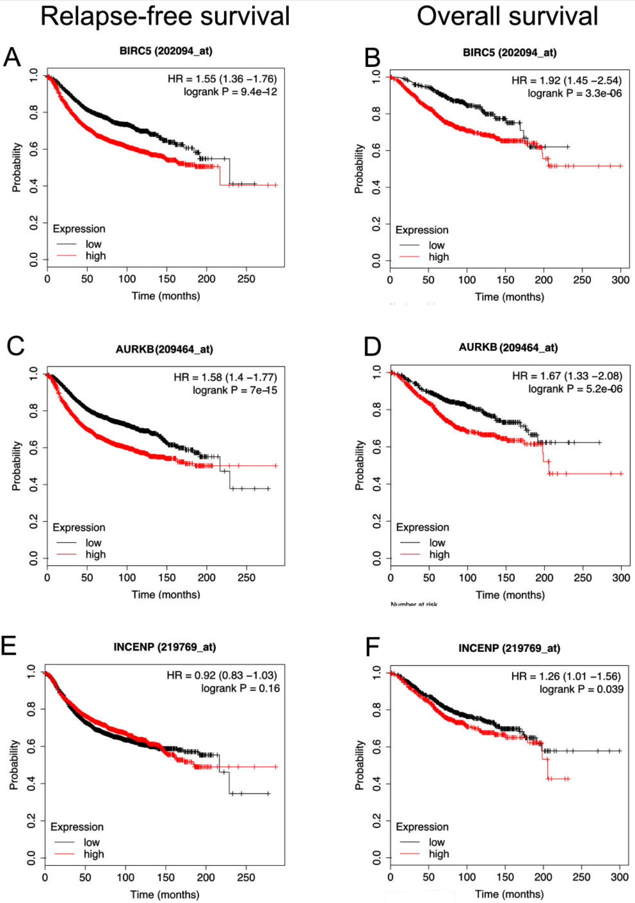

Supplement: Supplementary file 1 [file cells-09-00709-s001.zip › supple-proofreading/Figure S3.pdf]
