# Supplementary material for: Basal-Type Breast Cancer Stem Cells Over-Express Chromosomal Passenger Complex Proteins
Source: Cells. 2020 Mar 13;9(3):709. doi: 10.3390/cells9030709 (PMC7140627; doi:10.3390/cells9030709)
Supplement: Supplementary file 1 [file cells-09-00709-s001.zip › supple-proofreading/Table S1.pdf]

Basal-type Breast Cancer Stem Cells over-express chromosomal passenger complex proteins. Angela Schwarz-Cruz y Celis, et. al.

Supplementary Table 1. Primers used in the study

Name	Forward sequence (5'-3')	Reverse sequence (5'-3')
SDHA[1]	GGACCTGGTTGTCTTTGGTC	CCAGCGTTTGGTTTAATTGG
AURKB	TTGGACCCCAGGCTCCATCT	TGTGAAGTGCCGCGTTAAGA
INCENP	GTGCAGAGGAACCAGATGCT	CCTTCTCGACGAAGCTGCAC
KRT16	AGGAGATTGCCACCTACCGC	GACGGCTCGAAGAGGACGAG
KRT14	CTTCGCACCAAGAACTGAGG	TGTATTGATTGCCAGGAGGGG
IFI44L	CCTCTTCTAACAAACCCATGC TT	ACTGATTTGCCCCAGTAACCT
IFI44F	CCACCGAGATGTCAGAAAGAG	TGGTACATGTGGCTTTGCTC
H2AFZ	IDT Prime Time Hs.PT.58.895260.g	
BIRC5	IDT assay Hs.PT.56a.3412462	

1. Lemma, S., Avnet, S., Salerno, M., Chano, T. & Baldini, N. Identification and Validation of Housekeeping Genes for Gene Expression Analysis of Cancer Stem Cells. *PLoS One* **11**, e0149481 (2016).
